# In vitro antifungal susceptibilities of six antifungal drugs against clinical *Candida glabrata* isolates according to EUCAST

**DOI:** 10.18502/CMM.6.2.2692

**Published:** 2020-06

**Authors:** Mahnaz Fatahinia, Marzieh Halvaeizadeh, Ali Zarei Mahmoudabadi, Elham AboualiGalehdari, Neda Kiasat

**Affiliations:** 1 Infectious and Tropical Diseases Research Center, Health Research Institute, Ahvaz Jundishapur University of Medical Sciences, Ahvaz, Iran; 2 Department of Medical Mycology, School of Medicine, Ahvaz Jundishapur University of Medical Sciences, Ahvaz, Iran

**Keywords:** Ahvaz, Antifungal susceptibility test, *Candida glabrata*

## Abstract

**Background and Purpose:**

*Candida glabrata* is the second cause of candidiasis. The mortality rate of *C. glabrata* infections is about 40%; accordingly, it may be life threatening, especially in immunocompromised hosts. Regarding this, the current study was conducted to evaluate the regional patterns of the antifungal susceptibility of clinical *C. glabrata*isolated from the patients referring to the health centers located in Ahvaz, Iran

**Materials and Methods:**

In this study, a total of 30 clinical strains of *C. glabrata* isolates were recovered from different body sites (i.e., vagina, mouth, and urine). Phenotypic characteristics and molecular methods were used to identify the isolates. The minimum inhibitory concentration (MIC) was determined according to the European Committee on Antimicrobial Susceptibility Testing

**Results:**

Our findings demonstrated that 20%, 80%, and 6.7% of the isolates were resistant to amphotericin B, terbinafine, and posaconazole, respectively, while all the isolates were found to be fluconazole susceptible dose dependent and susceptible to voriconazole and caspofungin

**Conclusion:**

Our study suggested that voriconazole had high potency against *C. glabrata* isolates. Consequently, this antifungal agent can be an alternative drug in the treatment of resistant patients. These results can be helpful for the successful treatment of patients in different regions

## Introduction

in the past several decades, the prevalence of fungal infections, especially candidiasis, has been on a growing trend, which is often related to the immunological status of patients. Candidiasis is a fungal infection caused by genus *Candida*. This yeast not only causes disease in people with immune defects but also leads to an infection in healthy people [ 1 - 3]. *Candida* yeasts are settled as a normal mycoflora in the human mouth, gastrointestinal tract, and vagina, as well as in the environment [ 4 , 5].

*Candida albicans* is the most common causative agent of candidiasis; however, over the past decades, studies have shown an increasing prevalence of non-*albicans Candida* species, such as *C. glabrata*, *C. tropicalis*, *C. parapsilosis*, and *C. krusei* [ 6, 7]. *Candida glabrata* is the second causative agent of candidiasis, including candidemia, invasive candidiasis, oral candidiasis, urinary candidiasis, and vulvovaginal candidiasis [ 8 - 10]. The mortality rate of *C. glabrata* infections is about 40%; therefore, this species of *Candida* may be life threatening, especially in immunocompromised hosts [ 11, 12]. Regarding this, the diagnosis and treatment of *C. glabrata* infections are of paramount importance. However, this yeast presents intrinsic and acquired resistance to azole antifungals and may develop multi-drug resistance to other drugs.

Recently, researchers have reported that resistance to echinocandins is increasing, especially in fluconazole-resistant isolates. Hence, the treatment of the infections caused by *C. glabrata* remains a clinical challenge [ 13 - 16 ]. Accordingly, antifungal susceptibility testing is significant for the management of patients with *C. glabrata* infection. The European Committee on Antimicrobial Susceptibility Testing (EUCAST) and the Clinical and Laboratory Standards Institute (CLSI) have established standard methods based on minimum inhibitory concentration (MIC) in order to develop routine commercial drug sensitivity tests in the clinical laboratory [ 17]. Given the insufficiency of information about susceptibility and resistance to available drugs against *C. glabrata* in Iran, the objective of this study was to evaluate the antifungal susceptibility and resistance of *C. glabrata* isolated from patients referring to the health centers located in Ahvaz, Iran.

## Materials and Methods

**Sample collection**

This experiment was financially and ethically approved by the Research Deputy of Jundishapur University of Medical Sciences, Ahvaz, Iran (IR.AJUMS.REC.1392/ 907). A total of 30 isolates of *C. glabrata* were collected from different sites, such as the vagina (n=15, 50%), urinary tract (n=13, 43.3%), and mouth (n=2, 6.6%), in the patients referring to the health care centers in Khuzestan Province, Iran, from 2013 to 2016. All isolates were kept in sterile water at room temperature [ 18- 20].

**Classical identification of fungi**

Initially, all *Candida* isolates were confirmed by phenotypical characteristics as follows:

Evaluation of color on CHROMagar media (CHROMagar^TM^ *Candida*, France)

Absence of chlamydoconidia and hyphae on Corn Meal Agar media (Lifoilchem, Italy) with Tween 80 [ 21].

**Molecular identification**

**DNA extraction**

For the purpose of DNA extraction, the isolates were grown on Sabouraud dextrose agar media (Lifoilchem, Italy) overnight at 37℃. Subsequently, the colonies were transferred to 250 µl of sterile distilled water. The yeast was incubated at 100°C for 20 min and then centrifuged at 4°C for 10 min in 14,000 rpm. The supernatants of the isolates were collected into a new microtube. The DNA samples were measured by a nanodrop spectrophotometer (Thermo Scientific^TM^ NanoDrop^TM^ One Spectrophotometer) [ 22].

**Polymerase chain reaction amplification**

**Figure 1:**
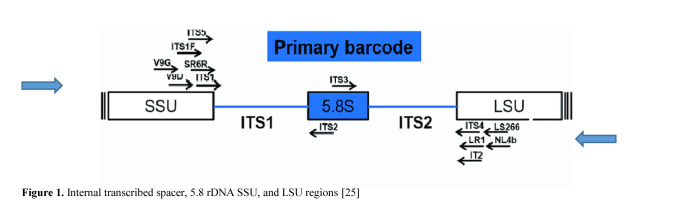
Internal transcribed spacer, 5.8 rDNA SSU, and LSU regions [ 25]

 The PCR identification of *C. glabrata* was carried out by both internal transcribed spacer (ITS) and 5.8 rDNA region, as well as the partials of SSU and LSU using universal primers V9G (5′ TTACGTCCCTGC CCTTTGTA 3′) and LS266 (5′ GCATTCCCAAACAACTCGACTC 3′). The target sequences were amplified with a cycle of 5 min at 95°C for primary denaturation, followed by 35 cycles at 95°C (30 sec), 58°C (30 sec), and 72°C (60 sec), and a final extension at 72°C for 10 min (Analytik Jena Thermocycler). Finally, the PCR products were run on 1% gel agarose to detect target fragment band with an approximate size of 1266 bp. As shown in Figure 1, ITS1 and ITS2 can be amplified with different primers. This study involved the amplification of the ITS1, 5.8S, ITS2, and parts of SSU and LSU, which is the larger fragment using the V9g and Ls266 primers (molecular weight of 1266 bp). In cases where the fragment sequence is performed by ITS1 and ITS4 primers as internal primers, the smaller region (molecular weight of 791 bp) is blasted, thereby facilitating more accurate identification [ 23 , 24]. 

**Anti-Fungal Susceptibility Testing**

The MIC was determined according to the EUCAST (version 9.0), which is valid based on the 2018-02-12 reference document [ 26]. Resazurin-based colorimetric assay (Sigma-Aldrich, Germany) was used for reading the MIC results [ 27]. The antifungal susceptibility of 30 *C. glabrata* isolates was assessed against amphotericin B (Sigma-Aldrich, Germany), fluconazole (Serva, USA), voriconazole (Sigma-Aldrich, Germany), posaconazole (Sigma-Aldrich, Germany), caspofungin (Sigma-Aldrich, Germany), and terbinafine (Sigma-Aldrich, Germany). In the first stage of AFST, antifungal agents were diluted with RPMI 1640 medium (Bio IDEA, Iran) and 0.01% of resazurin. The starting concentrations of fluconazole, voriconazole, posaconazole, caspofungin, terbinafine, and amphotericin B were 0.125-16, 0.004-1, 0.064-8, 0.016-2, 1-256, and 0.064-8 µg/ml, respectively. 

Each antifungal drug was attenuated by serial dilution with eight microtubes. Yeast suspensions were prepared from 24-hour fresh cultures of organisms. The percentage of optical absorbance was detected using a spectrophotometer at a wavelength of 530 nm in the range of 0.09-0.13. The final concentration of suspensions was determined as 1-5×10^6^ CFU/ML (0.5 McFarland standard), which was diluted with distilled sterile water at a ratio of 1:10.

Minimum inhibitory concentration is defined as the lowest concentration of the antifungal drug inhibiting 

the visible growth. Accordingly, MIC50 and MIC90 are defined as the lowest concentrations of the antifungal drugs that inhibit 50% and 90% of microorganism growth, respectively. Epidemiological cut-off values (ECV) are defined for the drugs having no specified clinical breakpoint (CBP). The ECV is determined when there is an overlap between wild type and non-wild type populations. A microorganism is defined as the wild type when it lacks intrinsic and acquired resistance (wild type and non-wild type strains). There are no clinical breakpoints for itraconazole, posaconazole, and voriconazole against *C. glabrata* microorganisms in the EUCAST guidelines [ 28, 29]. However, the MIC range, geometric mean, MIC^50^, and MIC^90^ have been defined for the isolates. 

In addition, the EUCAST guideline has not defined breakpoints for caspofungin and terbinafine. Therefore, caspofungin drug was interpreted based on the CLSI guideline, and terbinafine was analyzed according to a previous study [ 30, 31]. In the current study, *Candida krusei*Candida krusei ATCC 6258 and *C. parapsilosis* ATCC 22019 were selected as the standard strains. In terms of resistance to six drugs, the isolates were divided into several clusters using BioNumericsTM software (version 7.6, Applied Maths, License period: valid from 11/October/2018 until 10/November/2018; License string: 2KCN-45RP-DND7-47WW-FVHP-UV2M).

## Results

**Figure 2:**
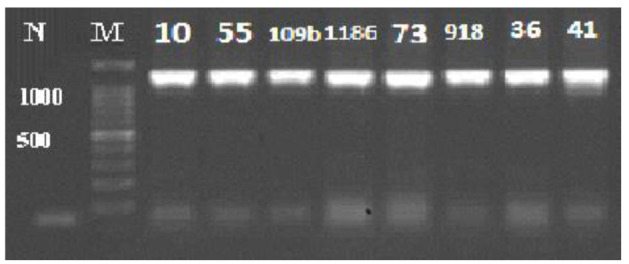
Electrophoresis of polymerase chain reaction products of the ITS gene region of *Candida glabrata* isolates using primers V9G and LS266 (sample numbers in order: 10, 55, 109b, 1186, 73, 918, 36, and 41).

All 30 samples were collected from patients based on the morphological characteristics that appeared on the chromogenic medium in white to pink-purple color. The suspected isolates were negative in terms of chlamydoconidia and hyphae production. Finally, *C. glabrata* isolates were confirmed by the amplification of the ITS gene region using primers V9G and LS266 (Figure 2). A summary of the activity of six antifungal agents against *C. glabrata* isolates is presented in Table 1. Our findings demonstrated that the non-wild type isolates showed 6.7% and 20% resistance to posaconazole (ECV>1) and amphotericin B (CBP>1), respectively. Our results showed that all isolates were susceptible dose dependent to fluconazole with a CPB of > 32 and had 100% sensitivity to voriconazole with an ECV of > 1 (wild type strains). 

(sample numbers in order: 10, 55, 109b, 1186, 73, 918, 36, and 41).

**Table1:** Antifungal susceptibility results of *Candida glabrata*.

Drug	MIC range	MIC_50_	MIC_90_	MIC_GM_	R non-WT n (%)	S N (%)	SDD N (%)
Amphotericin B	0.25-4	0.5	4	0.74055	6 (20)	24 (80)	0
Fluconazole	0.5-16	4	8	4.59479	0	0	30 (100)
Voriconazole	0.004-0.5	0.64	0.25	0.06784	0	30 (100)	NA
Posaconazole	0.032-4	0.032	0.25	0.06508	2 (6.7)	28 (93.3)	NA
Caspofungin	0.032-1	0.5	0.5	0.4458	NA	30 (100)	NA
Terbinafine	0.5-256	32	256	31.03335	24 (80)	6 (20)	NA

GM: geometric mean; R: resistant; non-WT: non-wild type, S: susceptible; SDD: susceptible dose dependent; NA: not available.

The MIC results for caspofungin and terbinafine were 0.032-1 and 0.5-256 µg/ml, respectively, with undefined ECV. The MICs_GM_ against all isolates were 0.4458, 0.06784, 0.74055, 0.06508, 31.03335, and 4.59479 µg/mL for caspofungin, voriconazole, amphotericin B, posaconazole, terbinafine, and fluconazole, respectively. The lowest MIC_50_ and MIC_90_ values were found for posaconazole, while the highest MIC_50_ and MIC_90_ were observed for fluconazole and terbinafine, which are summarized in Table 2. 

**Table2:** Differences in the minimum inhibitory concentration of isolates based on gender and source of isolates.

Number	Minimum inhibitory concentration								
	Collection number	Gender	Source	CASP	POS	AMB	VCZ	FCZ	TER
1	*C. glabrata (1128)*	F	Vagina	0.5	0.032	0.5	0.004	1	32
2	*C. glabrata (1131)*	F	Vagina	0.5	0.064	4	0.064	8	32
3	*C. glabrata (1134)*	F	Vagina	0.5	0.25	4	0.25	8	64
4	*C. glabrata (1158)*	F	Vagina	0.5	0.032	0.5	0.125	1	0.5
5	*C. glabrata (1162)*	F	Vagina	0.5	0.032	1	0.064	8	64
6	*C. glabrata (1179)*	F	Vagina	0.5	0.032	0.5	0.032	4	32
7	*C. glabrata (1186)*	F	Vagina	0.5	0.064	4	0.125	8	64
8	*C. glabrata (81b)*	F	Vagina	0.5	0.032	0.5	0.016	4	8
9	*C. glabrata (109b)*	F	Vagina	0.5	2	4	0.25	16	256
10	*C. glabrata (232)*	F	Vagina	1	0.032	0.5	0.004	4	128
11	*C. glabrata (kia2)*	F	Vagina	1	0.125	1	0.016	8	128
12	*C. glabrata (172)*	F	Vagina	1	0.032	1	0.016	8	128
13	*C. glabrata (73)*	F	Vagina	0.5	0.032	0.5	0.064	8	16
14	*C. glabrata (74)*	F	Vagina	0.5	0.25	1	0.125	8	256
15	*C. glabrata (918)*	F	Vagina	0.5	0.032	0.5	0.25	2	8
16	*C. glabrata*	M	Oral	0.25	0.125	0.5	0.125	8	128
17	*C. glabrata (1)*	M	Oral	0.5	0.032	2	0.125	16	16
18	*C. glabrata (4)*	M	Urine	0.032	0.032	0.5	0.064	4	32
19	*C. glabrata (5)*	M	Urine	0.5	4	2	0.5	16	256
20	*C. glabrata (8)*	M	Urine	0.5	0.032	0.5	0.25	4	8
21	*C. glabrata (18)*	M	Urine	0.5	0.032	0.5	0.032	2	2
22	*C. glabrata (35)*	M	Urine	0.5	0.25	0.5	0.125	4	64
23	*C. glabrata (36)*	M	Urine	0.5	0.032	0.5	0.125	4	16
24	*C. glabrata (39)*	M	Urine	0.5	0.032	0.5	0.064	8	64
25	*C. glabrata (41)*	M	Urine	0.5	0.032	0.25	0.032	0.5	64
26	*C. glabrata (42)*	M	Urine	0.5	0.032	0.5	0.032	4	16
27	*C. glabrata (43)*	M	Urine	0.125	0.032	0.25	0.064	4	0.5
28	*C. glabrata (55)*	M	Urine	0.5	0.032	0.25	0.25	8	16
29	*C. glabrataM*	Urine	0.5	0.25	0.5	0.125	4	16
30	*C. glabrata (10)*	M	Urine	0.25	0.032	0.5	0.064	4	256

POSA: posaconazol, CASP: caspofungin, AMB: amphotericin B, VOR: voriconazole, TER: terbinafine, FCZ: fluconazole.

**Figure 3:**
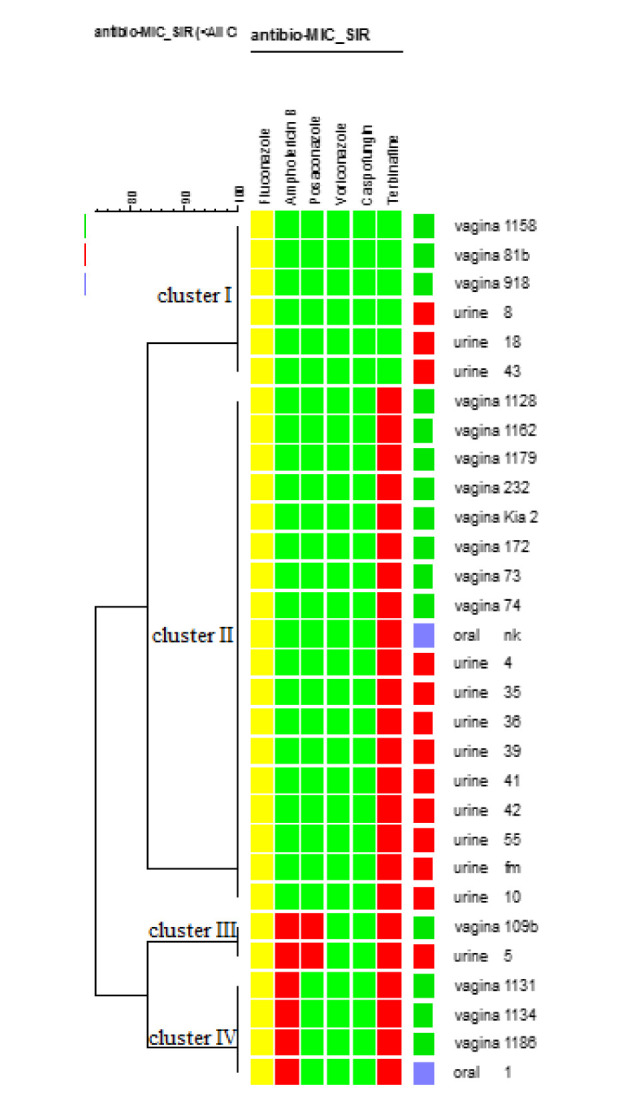
Dendrogram of susceptibility to six antifungal agents (i.e., amphotericin B, fluconazole, voriconazole, posaconazole, caspofungin, and terbinafine) among 30 *Candida glabrata* isolates using the BioNumericsTM software (version 7.6, Applied Maths) showing the antifungal drug resistance pattern following microdilution assay (Epidemiological cut-off values and breakpoints were used for interpretation according to the EUCAST 2018 guideline that was divided to four clusters. Yellow, green, and red colors indicate susceptible dose-dependent, susceptible, and resistant fungi, respectively.).

Figure 3 depicts a dendrogram based on drug resistance profile in which the isolates of *C. glabrata*

are divided into four clusters. Cluster I is composed of isolates from the urine and vagina non-resistant to the drug, and cluster II has 18 isolates from the vagina, mouth, and urine resistant to terbinafine. In addition, cluster III consists of amphotericin B-resistant isolates and a posaconazole-resistant isolate from the vagina and urine, and cluster IV includes amphotericin B- and terbinafine-resistant isolates from the vaginal and oral sources. Generally, 20% of the isolates showed resistance to 2-3 antifungal agents and were classified as multidrug-resistant tuberculosis in the current research (Figure 3). 

## Discussion

It seems that the issue of drug resistance gradually becomes more significant in the field of therapy and poses challenges in terms of treatment cost and response to the drug for patients and hospitals. *Candida glabrata* isolates are increasingly becoming resistant to azoles, echinocandins, and polyenes [ 32 - 34]. The purpose of this paper was to identify the regional pattern of *C. glabrata* antifungal susceptibility in the samples collected from patients visiting Ahvaz health centers.

As mentioned earlier, the samples had been collected in previous studies (references were cited) and were only molecularly confirmed in this study (Figure 2). In the present research, based on the EUCAST guideline, the highest resistance rate to amphotericin B (20%) was observed at the MIC range of 0.25-4 µg/mL, MIC_90_ of 4µg/mL, MIC_50_ of 0.5 µg/mL, and geometric mean MIC of 0.44 µg/mL, which is similar to the previous reports [ 35, 36]. This study indicated that 6.7% of the isolates were resistant (non-wild type) to posaconazole with an MIC range of 0.032-4 µg/mL, which is consistent with the results obtained by Badie *et al*. [ 37]. On the other hand, in this experiment, 93% and 100% of the isolates were susceptible to posaconazole and voriconazole, respectively, with the lowest MIC_GM_ and MIC range, compared to those of other antifungal drugs (i.e., fluconazole, terbinafine, amphotericin B, caspofungin). These results indicate that voriconazole drug is an antifungal active against *C. glabrata* isolates.

This study showed that terbinafine had the highest resistance and MIC_GM_ and that it cannot be an effective drug against *C. glabrata* species. These results are in agreement with those of other studies [ 30 , 38, 39]. In addition, *C. glabrata* isolates were found to be susceptible to fluconazole, which is in line with the results reported by Morales-Lopez *et al*. but different from the data obtained by other studies, such as those conducted by Amirrajab *et al*. and Badiee *et al*. [ 35 , 37 , 40- 44]. It seems that these differences are due to the source of isolation, exposure of patients to high antifungal doses, and clinical status of patients. 

Based on the results of the current study, caspofungin was an effective drug tested against *C. glabrata* with the MIC range of 0.032-1 µg/mL, MIC_90_ of 0.5 µg/mL, MIC_50_ of 0.5 µg/mL, and geometric mean MIC of 0.44 µg/mL that is in agreement with the results reported by Labbe *et al*. and other researchers [ 35, 37, 45, 46]. Our study showed that MIC_90_ values for fluconazole, posaconazole, and voriconazole were significantly lower than those obtained by Espinel-Ingroff *et al*. [ 47]. This discrepancy could be attributed to the misdiagnosis of fungal diseases, as well as the high cost and unavailability of some drugs, such as posaconazole, in Iran.

According to the sexually transmitted diseases treatment guidelines (2015), a general treatment is not known for vaginal candidiasis with *C. glabrata* yeast. Therefore, the first line of recommended treatment is a non-fluconazole azole regimen (oral or topical), which was confirmed by our study. Voriconazole drug is potentially active against *C. glabrata* [ 48]. It was preferred to discuss patients' demographics, age, underlying diseases, and consumed medications. However, as mentioned earlier, the samples had been collected in previous studies, and access to patient information was not possible.

## Conclusion

Voriconazole can be an alternative drug when patients do not respond to another azole class of drugs. The advent of resistant isolates to amphotericin B and posaconazole may become a serious therapeutic problem in the world. This highlights the importance of performing antifungal susceptibility tests. Therefore, this study suggests the implementation of annual evaluations in every province of Iran to assess the resistance of *C. glabrata* to antifungals with the aim of making a reliable decision to control and successfully treat *C. glabrata* infections. Future studies are recommended to replicate results in a larger collection of *C. glabrata* isolates.
